# Inhibition of p38 MAPK Signaling Regulates the Expression of EAAT2 in the Brains of Epileptic Rats

**DOI:** 10.3389/fneur.2018.00925

**Published:** 2018-10-29

**Authors:** Zhang Yang, Jing Wang, Changyin Yu, Ping Xu, Jun Zhang, Yan Peng, Zhong Luo, Hao Huang, Junwei Zeng, Zucai Xu

**Affiliations:** ^1^Department of Neurology, Affiliated Hospital of Zunyi Medical University, Zunyi, China; ^2^Department of Neurology, Affiliated Hospital of Guizhou Medical University, Guiyang, China; ^3^Department of Prevention and Health Care, Affiliated Hospital of Zunyi Medical University, Zunyi, China; ^4^Department of Physiology, Zunyi Medical University, Zunyi, China; ^5^Key Laboratory of Brain Science, Zunyi Medical University, Zunyi, China

**Keywords:** epilepsy, glutamic acid, SB203580, excitatory amino acids, p38 mitogen-activated protein kinase

## Abstract

Seizures induce the release of excitatory amino acids (EAAs) from the intracellular fluid to the extracellular fluid, and the released EAAs primarily comprise glutamic acid (Glu) and asparaginic acid (Asp). Glu neurotransmission functions via EAA transporters (EAATs) to maintain low concentrations of Glu in the extracellular space and avoid excitotoxicity. EAAT2, the most abundant Glu transporter subtype in the central nervous system (CNS), plays a key role in the regulation of glutamate transmission. Previous studies have shown that SB203580 promotes EAAT2 expression by inhibiting the p38 mitogen-activated protein kinase (MAPK) signaling pathway, but whether SB203580 upregulates EAAT2 expression in epileptic rats is unknown. This study demonstrated that EAAT2 expression was increased in the brain tissue of epileptic rats. Intraperitoneal injection of a specific inhibitor of p38 MAPK, SB203580, reduced the time to the first epileptic seizure and attenuated the seizure severity. In addition, SB203580 treatment increased the EAAT2 expression levels in the brain tissue of epileptic rats. These results suggest that SB203580 could regulate epileptic seizures via EAAT2.

## Introduction

Epilepsy is a global public health problem. In fact, there are ~70 million epilepsy patients worldwide (1), and more than 90% of these patients are located in low- and middle-income countries. According to statistical data, epilepsy accounts for 0.7% of the global burden of disease. Repeated epileptic seizures have negative effects on mood, behavior, personality, cognition, movement, and sleep and can even increase mortality in patients with epilepsy (2).

Imbalances in the levels of central nervous system (CNS) neurotransmitters are important mechanisms in epilepsy. Epileptic seizures can increase the release of excitatory amino acids (EAAs), which primarily include glutamic acid (Glu) and asparaginic acid (Asp), from inside the cell to the outer environment (3). Glu is the major excitatory neurotransmitter of the CNS, and abnormal expression levels of its receptor and transporter are closely related to the pathogenesis of epilepsy (4). Studies have shown that seizures in epileptic patients can abnormally increase the extracellular Glu and Glu receptor levels, and these increased levels of EAAs increase the number of CNS neurons in a state of excessive excitability (5). Through brain microdialysis, other scholars have shown that extracellular Glu levels continue to increase before and during seizures in epilepsy patients (3, 6); in addition, as demonstrated in a rat model of epilepsy, excessive increases in the extracellular Glu levels lead to toxic neuronal excitability and might eventually lead to neuronal death after seizures (7, 8). EAA transporter 2 (EAAT2) is distributed in the hippocampus and nerve cells of the prefrontal lobe in mammals. This transporter can reduce nerve cell injury by absorbing extracellular Glu and transporting it into cells, and this process reduces the Glu levels in the synaptic cleft and decreases the excitotoxicity of Glu to neurons and glial cells (9–12). In addition, there is a type of stress-activated protein kinase in the mammalian CNS through which mitogen-activated protein kinase (MAPK) can transmit extracellular signals to the nucleus (13). This kinase, p38 MAPK, is a member of the MAPK family, which plays an important role in cell survival, differentiation and development. However, activation of p38 MAPK in the CNS causes neuronal cell death (14). Studies have shown that in the CNS, the binding of Glu to the NMDA receptor can activate p38 MAPK and induce the apoptosis of hippocampal granule cells in the brain (15). In addition, Okamoto performed a genome-wide screen to show that p38 MAPK is overexpressed in the hippocampus of epileptic rats (16). Consequently, p38 MAPK might play a role in epilepsy. Additionally, a study by Liang et al. showed that activated δ opioid receptors potentially regulate the methyl ethyl ketone (MEK)-extracellular signal-regulated kinase (ERK)-p38 MAPK cascade transduction pathway to promote the expression of EAAT1 and EAAT2 in astrocytes and decrease the concentration of extracellular Glu (17). In summary, p38 MAPK, Glu and EAAT2 appear to be associated with epilepsy, and SB203580 competitively binds to ATP binding sites for the purpose of inhibiting p38MAPK, thereby effectively inhibiting some signal transduction induced by some inflammatory factors. However, whether the regulation of p38 MAPK is related to the expression of EAAT2 during epileptic seizures has not been reported in the literature. Therefore, this study assessed the changes in the epileptic seizure intensity and in the expression levels of EAAT2 in brain tissues induced by the p38 MAPK inhibitor SB203580 in a lithium chloride-pilocarpine-induced rat model of epilepsy.

## Materials and methods

### Establishment of the lithium chloride-pilocarpine-induced rat model of epilepsy

Male Sprague Dawley (SD) rats weighing ~180–220 g were purchased from the Experimental Animal Center of the Third Military Medical University [clean facility; license number: SCXK, (Yu) 2012-0005]. All animal experiments were performed in accordance with the Guidelines for Animal Experiments of the Chinese Academy of Medical Sciences and with approval from the Ethics Committee for Animal Care of the Third Military Medical University. The rats were maintained in an environment with a constant temperature (24 ± 2°C) and received pure drinking water and standard feed via conventional cage feeding. A total of 162 adult male SD rats were included in this study. Forty-seven were excluded (32 rats died and 15 failed). The rats were randomly divided into the following groups: the untreated control, the epilepsy group (0 h, 1 d, 3 d, 1 w, and 2 w), the SB203580 group, and the solvent control group (normal saline + 2% DMSO).

Randomly selected healthy adult male SD rats received an intraperitoneal injection of lithium chloride (127 mg/kg); 20 h later, the rats were intraperitoneally injected with atropine sulfate (1 mg/kg), and 30 min later, the rats were intraperitoneally injected with pilocarpine (50 mg/kg). Seizure behaviors after kindling were classified according to the standards of the Racine scale as follows: stage I, mouth and facial movements; stage II, head nodding; stage III, forelimb clonus; stage IV, rearing and stage V, rearing and falling. If the rats did not have a Racine score of IV-V after 30 min (18), single intraperitoneal injections of pilocarpine (10 mg/kg) were administered until seizure development. After the rats achieved status epilepticus (SE) and maintained this status for 45 min, they were administered an intraperitoneal injection of atropine sulfate (1 mg/kg) and diazepam (10 mg/kg) to terminate the seizure. The behaviors of the rats were monitored and recorded, and the Racine scores of the experimental group reached IV-V. The rats in the untreated control group received the same treatment with lithium chloride and atropine sulfate, but were administered saline instead of pilocarpine. The rats in the SB203580 group received an intraperitoneal injection of SB203580 (15 mg/kg; normal saline + 2% freshly prepared DMSO) (19) 30 min before the pilocarpine injections, and the rats in the solvent control group received the same concentration of DMSO as the SB203580 group.

### Western blotting

To collect brain tissue from each group of rats, the rats were anesthetized with 10% chloral hydrate (0.33 ml/100 g) by intraperitoneal injection. After administration of the anesthesia, the skull was rapidly removed and separated, and the brain tissue was removed. The bilateral hippocampus and the cortex of the adjacent temporal lobe were quickly separated from the bath, and the specimen was wrapped with tin foil, placed in a cryotube and stored in a liquid nitrogen tank. All the samples were homogenized in 30 mM Tris-HCl and 100 mM phenylmethylsulfonyl fluoride. The protein concentrations of the supernatants were measured using an Enhanced BCA Protein Assay Kit (Beyotime, Haimen, China) according to the manufacturer's instructions. Equal amounts of protein samples were run on SDS-PAGE gels (5% stacking gel; 10% separation gel) and transferred to polyvinylidene difluoride membranes. The PVDF membranes had been pretreated with methanol for ~5 min, and the filter paper and sponge were soaked into the electro-transfer buffer during storage. The gel was sandwiched between the sponges and filter paper and rolled with a roller to remove bubbles. The sandwich was electro-transferred for 4 h at 40 V, and the membrane was blocked in 5% skim milk at RT for 3–4 h. After this process, the PVDF membrane was removed and incubated overnight at 4°C with anti-EAAT2 (rabbit monoclonal antibody, 1:2,000 dilution, Abcam, ab203130) and then at 23°C for 2 h with HRP-tagged secondary antibody (1:1,000, Santa Cruz Biotechnology, CA, USA, sc-2004). Blots were developed using Super Signal West Pico Chemiluminescent HRP substrate (Rockford, IL, USA) according to the manufacturer's instructions, and the blot intensities were calculated using Quantity One software (Bio-Rad Laboratories, Hercules, CA, USA).

### Immunohistochemistry staining

The anesthetized animals were transcardially perfused with saline and then with 4% formaldehyde in phosphate buffer (pH 7.4) for 10 min. The brains were then carefully removed, post-fixed at 4°C in the formaldehyde solution for 24 h, dehydrated in a series of graded ethanol solutions and cleared in xylene. The samples were subsequently embedded in paraffin, and 4- to 5-μm-thick coronal sections were cut.Paraffin sections were deparaffinized in xylene and rehydrated in a graded series of ethanol solutions. After three 15-min washes with phosphate-buffered saline, the sections were incubated in 3% H_2_O_2_ for 15 min, heated (92–98°C) in 10 mmol/l boiling sodium citrate buffer (pH 6.0) for 20 min and blocked with normal goat serum (1:10) for 10 min (Gene Tech Inc., Shanghai, China).The sections were then incubated with anti-EAAT2 (rabbit monoclonal antibody, 1:200 dilution, Abcam, ab203130) at 37°C for 2 h, subjected to three 30-min washes with PBS, incubated with biotinylated goat anti-rabbit secondary antibody (Gene Tech Inc., Shanghai, China, abs20003) for 30 min and washes again. The sections were subsequently treated with ABC solution for 30 min at 37°C and washed three times with PBS (5 min each time). DAB coloring solution was then dropped onto the sections. As determined by observation using a microscope, when brownish yellow particles appeared, pure water was added to terminate the coloration reaction. The paraffin sections were rehydrated in a graded series of ethanol solutions and deparaffinized in xylene. An Olympus PM20 automatic microscope (Olympus, Osaka, Japan) and a TC-FY-2050 pathology system (Yuancheng Inc., Beijing, China) were used for image collection. If the cytoplasm was dyed a yellowish color, the sample was considered positive for EAAT2. For each sample, images of ten random visual fields at 20x magnification were selected for analysis. The HPIAS 1000 high-definition image analysis system (Qianping Image Technology Co., Ltd., Wuhan, China) was used to count the EAAT2-positive cells.

### Immunofluorescence staining

To confirm the location of EAAT2 in the neurons and astrocytes in the hippocampus after seizure, the immunoreactivity for EAAT2 in rats 3 d after seizure onset was observed by double-labeling immunofluorescence. Tissues sections were permeabilized with 0.5% Triton X-100 and incubated first with 10% goat serum (Zhongshan Golden Bridge, Inc., Beijing, China) for 30 min and then with rabbit anti-EAAT2 (1:200; Abcam, Cambridge, UK, ab203130) and mouse anti-GFAP antibodies (1:100; Santa Cruz Biotechnology, USA, sc-71143) overnight at 4°C. The next day, the sections were washed five times with phosphate-buffered saline (55 min total) and incubated in the dark with secondary antibodies (anti-rabbit-Alexa 405, green, Abcam, Cambridge, UK, ab169345; anti-rabbit-Alexa 488, blue, Boster, Inc., Wuhan, China, F06630-1; anti-rabbit-Alexa 549, red, Zhongshan Golden Bridge, Inc., Beijing, China, SC-549) at 1:100 dilution for 60 min. The sections were then counter-stained with DAPI (4′,6-diamidino-2-phenylindole, 1:10,000 dilution, Sigma-Aldrich, D9542) and PBS for 20 min, mounted and sealed with 50% glycerin, and the slides were then observed using a laser scanning confocal microscope at 40x magnification.

### Statistical methods

The data are expressed as the means ± standard deviations and were analyzed using SPSS version 18.0. The behavioral characteristics of the rats (seizure duration and Racine score) and the immunohistochemistry and Western blot results were compared by two-way ANOVA; *P* < 0.05 was used to indicate a significant difference.

## Results

### Effects of SB203580 on the duration of the first seizure and racine scores in rats

**Figure 1:**
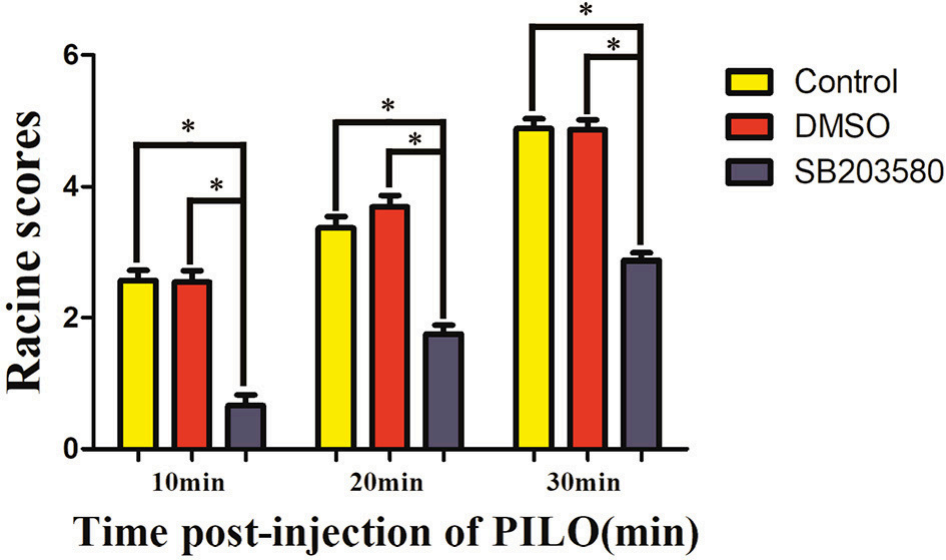
Racine scores of different groups at different time points after the pilocarpine injection (min). ^*^P < 0.05 compared with the control (epilepsy) and DMSO groups.

**Figure 2:**
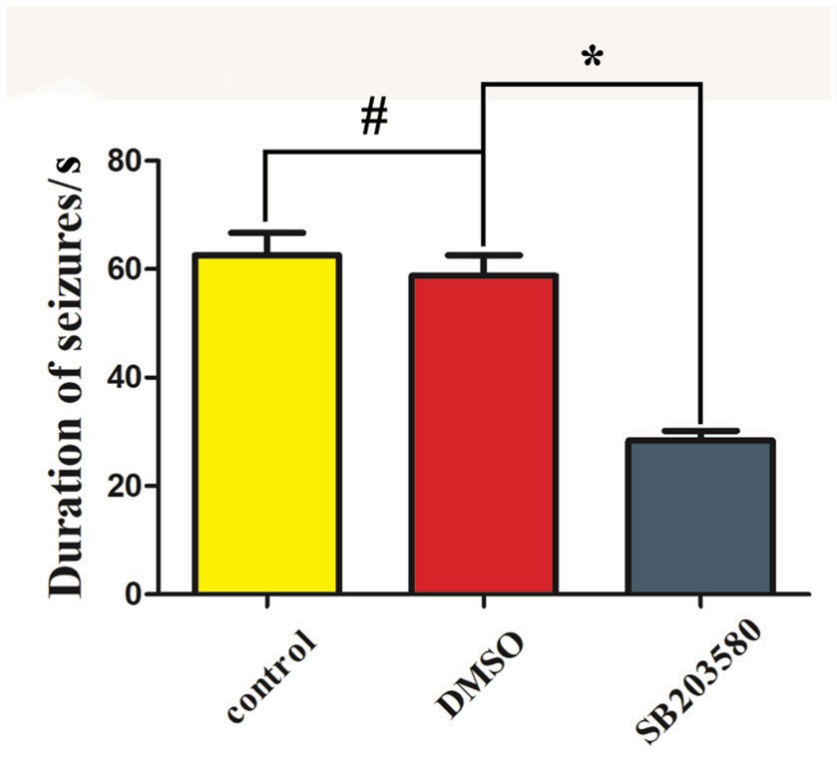
Duration of the first seizure episode in the different groups. ^*^P < 0.05 compared with the SB203580 group; #*P* > 0.05, compared with the control (epilepsy) groups.

The Racine scores of the epilepsy, solvent control and SB203580 groups were 2.57 ± 0.48, 2.55 ± 0.52, and 0.66 ± 0.48, respectively, 10 min after the intraperitoneal injection of pilocarpine; 3.37 ± 0.50, 3.96 ± 0.55, and 1.75 ± 0.43, respectively, at 20 min after the intraperitoneal injection of pilocarpine; and 4.88 ± 0.46, 4.87 ± 0.45, and 2.87 ± 0.32, respectively, at 30 min after the intraperitoneal injection of pilocarpine. The duration of the seizures in the epilepsy, solvent control and SB203580 groups was 62.56 ± 9.22 s, 58.79 ± 8.41 s, and 28.35 ± 4.00 s, respectively. No significant differences in the Racine scores or seizure duration were found between the epilepsy and solvent control groups (*P* > 0.05). Compared with the epilepsy and solvent control groups, the Racine score in the SB203580 group was significantly decreased at the three tested time points after the pilocarpine injection, and the seizure duration was significantly shortened. The intraperitoneal injection of SB203580 significantly reduced the Racine scores and the duration of the first seizure in lithium chloride-pilocarpine-induced epileptic rats (*P* < 0.05); however, the effect of 2% DMSO on these parameters was not statistically significant (Figures 1, 2).

### Effects of seizure and SB203580 (a specific inhibitor of p38 MAPK) on EAAT2 expression in brain tissue

**Figure 3:**
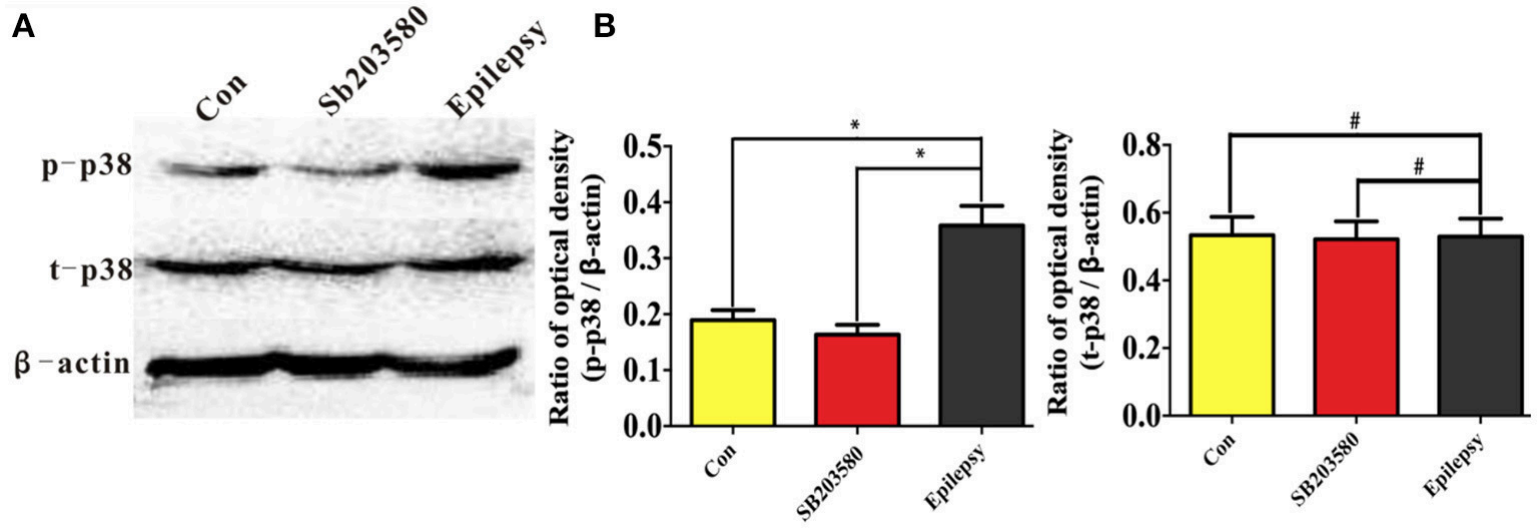
Hippocampal t-p38 and p-p38 expression in the different groups measured by Western blotting. Panel **(A)** shows the Western blotting image of temporal neocortex expression in different groups, and **(B)** shows the quantification of the data shown in the image in **(A)**.^*^P < 0.05 compared with the untreated control and SB203580 groups; #*P* < 0.05 compared with the untreated control and SB203580 groups.

A Western blot analysis was used to assess the expression levels of total and phosphorylated p38 protein (T-p38 and P-p38, respectively) in the untreated control, SB203580 and epilepsy groups. The immunoblot density ratios of T-P38 to the corresponding internal reference (β-actin) in the control, SB203580, and epilepsy groups were 0.195 ± 0.012, 0.174 ± 0.015, and 0.376 ± 0.02, respectively. The expression of T-P38 in the hippocampus of the epileptic rats was significantly increased (*P* < 0.05) compared with that in the untreated control and SB203580 rats. In addition, the immunoblot density ratios of P-p38 to the corresponding internal reference (β-actin) in the control, SB203580, and epilepsy groups were 0.538 ± 0.021, 0.517 ± 0.018, and 0.523 ± 0.015, respectively. No significant difference (*P* > 0.05) in P-p38 expression was found between the untreated control group and the SB203580 group (Figure 3).

**Figure 4:**
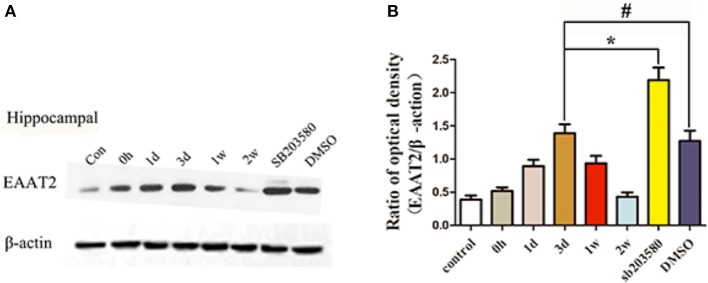
Hippocampal EAAT2 expression in the different groups measured by Western blotting. Panel **(A)** shows the Western blotting image of hippocampal expression in the different groups, and **(B)** shows the quantification of the data in the image shown in **(A)**. ^*^P < 0.05 for the comparison of the SB203580 group with the 3-d group; ^#^*P* > 0.05 for the comparison of the DMSO group with the 3-d group.

EAAT2 was expressed at basal levels in the hippocampus of the untreated control group, and the immunoblot density ratio of EAAT2 to the corresponding internal reference (β-actin) was 0.39 ± 0.15. In the epilepsy group, the immunoblot density ratios of EAAT2 to β-actin were 0.52 ± 0.13, 0.89 ± 0.24, 1.39 ± 0.33, 0.93 ± 0.27, and 0.43 ± 0.16 at different times. At the first three time points after the onset of seizure, the EAAT2 expression levels were significantly increased (*P* < 0.05) compared with those of the untreated control group, and EAAT2 expression peaked at day 3. At 1 and 2 w after seizure onset, the EAAT2 expression levels began to decrease to the normal levels. According to these results, the highest expression level of EAAT2 occurred 3 d after the onset of epileptic seizures. Therefore, 3 d after seizure onset was selected as the time point for measuring the effects of the p38 MAPK inhibitor SB203580. SB203580 was injected intraperitoneally 30 min before the seizure model was induced, and brain tissue proteins were extracted for Western blot analyses. In the rat hippocampus, the immunoblot density ratios of EAAT2 to β-actin in the SB203580 and solvent control groups were 2.19 ± 0.46 and 1.27 ± 0.46, respectively. The Western blot results showed that the expression level of EAAT2 in the SB203580 group at 3 d was significantly higher than that in the epilepsy group (*P* < 0.05). At 3 d, no significant difference in EAAT2 expression was found between the solvent control group and the epilepsy group (*P* > 0.05) (Figure 4).

**Figure 5:**
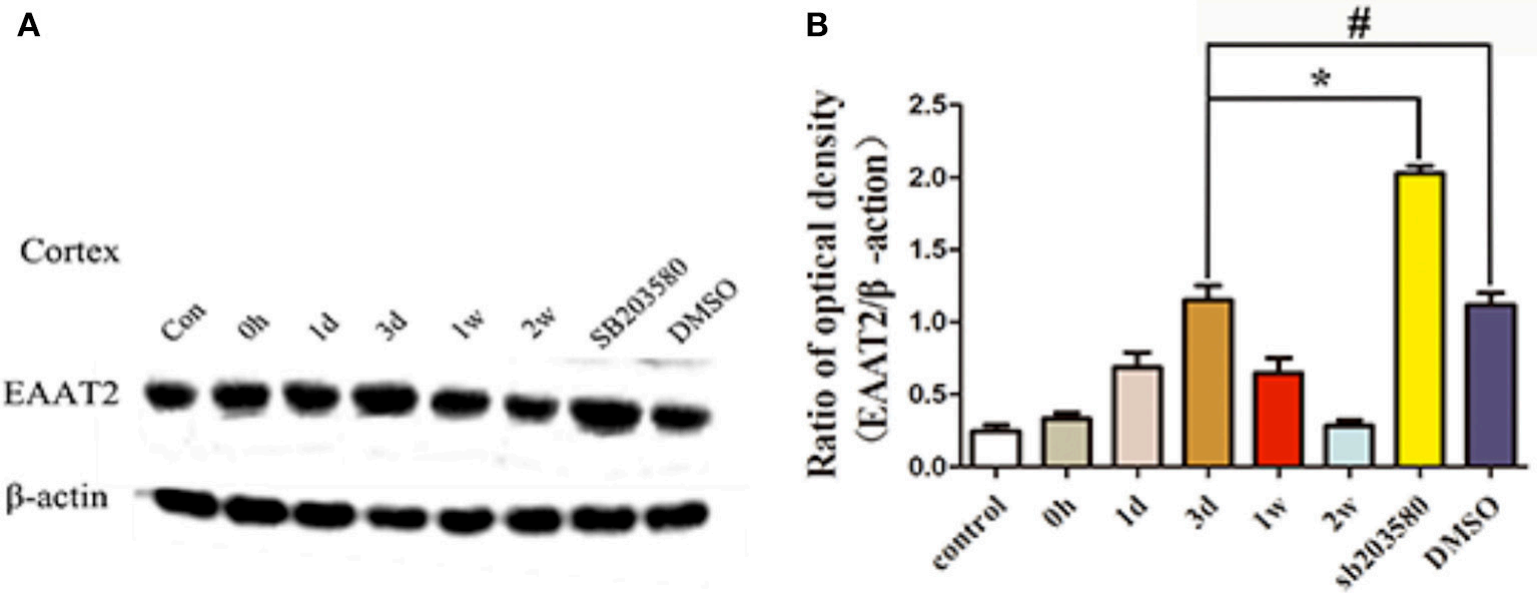
Temporal neocortex EAAT2 expression in the different groups measured by Western blotting. Panel **(A)** shows the Western blotting image of temporal neocortex expression in the different groups, and **(B)** shows the quantification of the data in the image shown in **(A)**. ^*^ P < 0.05 for the comparison of the SB203580 group with the 3-d group; ^#^*P* < 0.05 for the comparison of the DMSO group with the 3-d group.

The Western blot results showed that EAAT2 was expressed at basal levels in the temporal neocortex of the rats in the untreated control group, and the immunoblot density ratio of EAAT2 to β-actin was 0.25 ± 0.1. In the epilepsy group, the immunoblot density ratios of EAAT2 to β-actin at various time points were 0.33 ± 0.09, 0.69 ± 0.25, 1.15 ± 0.25, 0.65 ± 0.26, and 0.28 ± 0.09. Specifically, From 0 h to 3 d after seizure onset, the EAAT2 expression levels were significantly increased (*P* < 0.05) in the epilepsy group compared with the untreated control group, and EAAT2 expression peaked 3 d after seizure onset. At 1 and 2 w after seizure onset, the EAAT2 expression levels began to decrease to the normal levels. These results show that the highest expression level of EAAT2 occurred 3 d after the onset of epileptic seizures. Therefore, 3 d after seizure onset was selected as the time point for measuring the effects of the p38 MAPK inhibitor SB203580. SB203580 was injected intraperitoneally 30 min before the model was induced, and brain tissue proteins were extracted for Western blot analyses. In the temporal neocortex of rats, the immunoblot density ratios of EAAT2 to β-actin in the SB203580 and solvent control groups were 2.03 and 1.12, respectively. The Western blot results showed that EAAT2 expression in the SB203580 group at 3 d after seizure onset was significantly higher than that in the epilepsy group (*P* < 0.05), and no significant difference in EAAT2 expression was found between the solvent control group and the epilepsy group at this time point (*P* > 0.05) (Figure 5).

These results suggest that using SB203580 to inhibit p38 MAPK can increase the expression levels of EAAT2 in the hippocampus and neocortex of epileptic rats.

### Hippocampal EAAT2 expression measured by immunohistochemistry and effect of SB203580 on its expression

**Figure 6:**
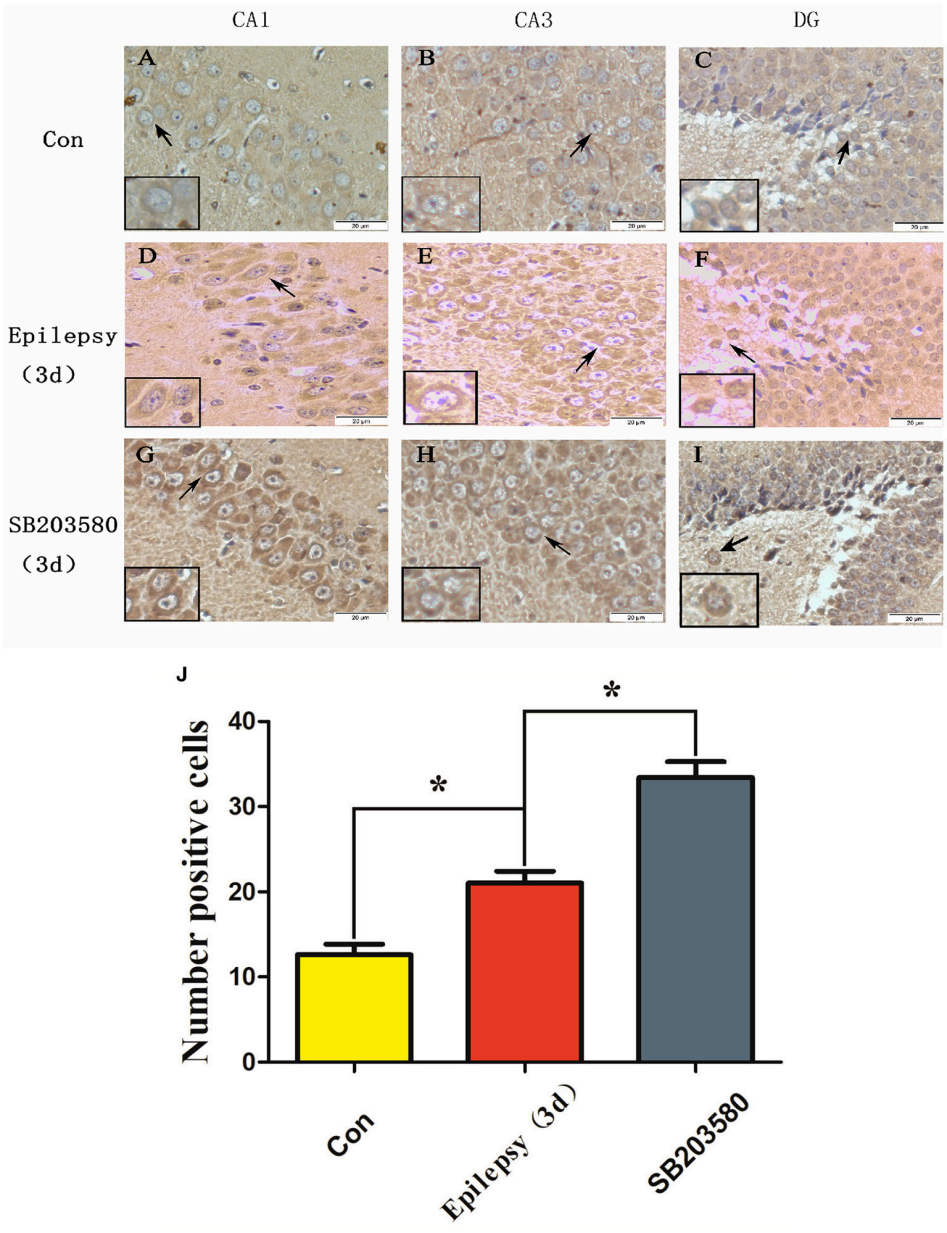
Immunohistochemical detection of EAAT2 expression in the hippocampus (scale: 20 μm). **(A,D,G)** show the CA1 hippocampal area; **(B,E,H)** show the CA3 hippocampal area; and **(C,F,I)** show the DG hippocampal area. Panel **(J)** shows the quantification of **(A–I)**. ^*^P < 0.05 compared with the untreated control.

The Western blot results showed that the protein expression levels of EAAT2 in the hippocampus of epileptic rats were significantly higher than those in the untreated control group, and the highest level of expression occurred 3 d after seizure onset. For this reason, 3 d after seizure was selected as the time point for measuring EAAT2 expression in the hippocampus by immunohistochemistry (Figure 6). The results showed that at 3 d, EAAT2 was mainly expressed in the neuronal cell membrane of the CA1, CA3 and DG hippocampal areas in the untreated control, seizure and SB203580 groups. In addition, we performed a semi-quantitative analysis of the number of EAAT2-positive cells. The number of EAAT2-positive cells in the seizure group at 3 d was significantly increased (*P* < 0.05) compared with that in the untreated control group. Moreover, the increase in the number of EAAT2-positive cells was significantly greater in the SB203580 group than in the seizure group at 3 d (*P* < 0.05).

### EAAT2 cellular localization in the hippocampus measured by immunofluorescence

**Figure 7:**
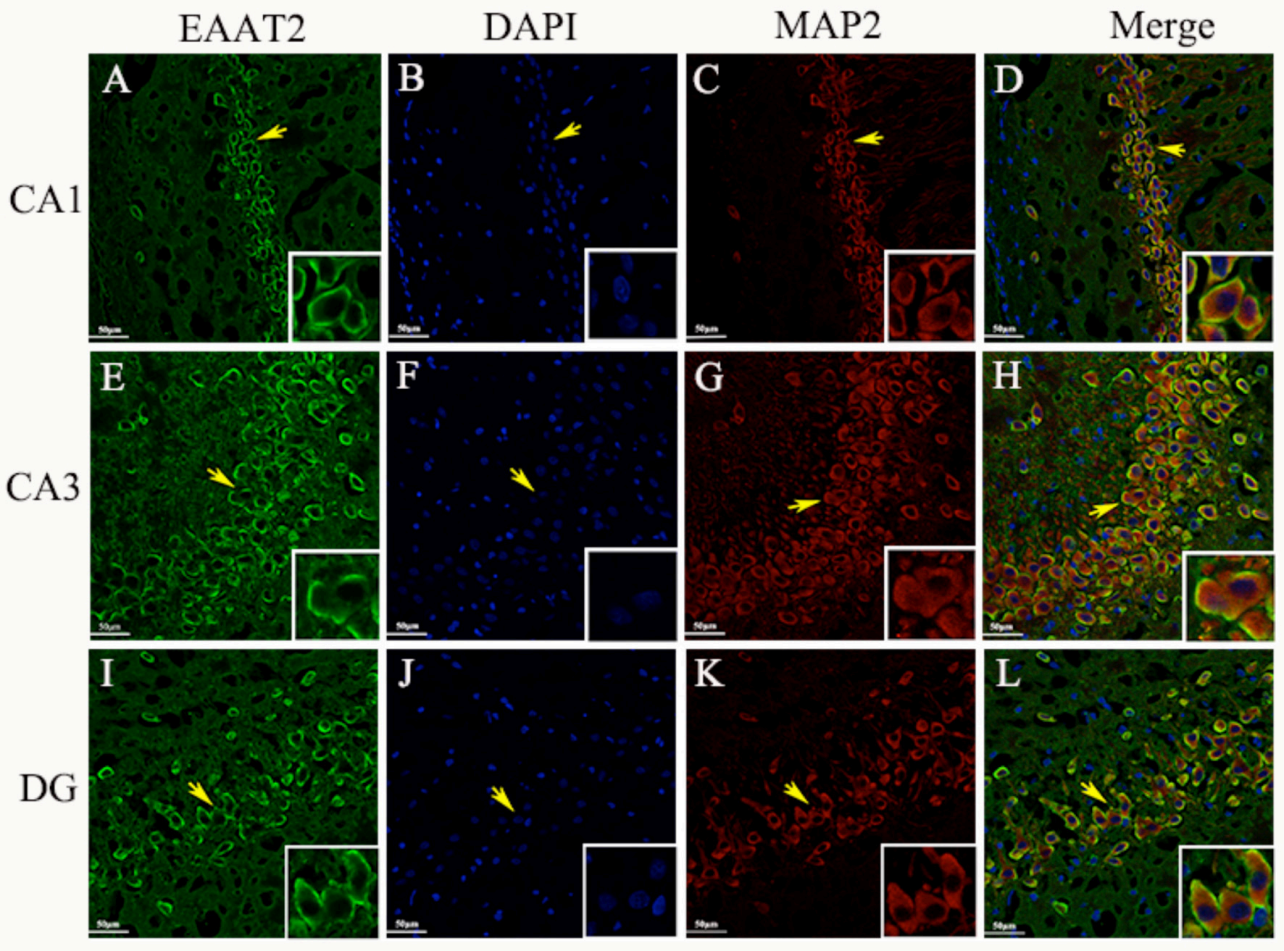
Neuronal cells **(A–L)**. EAAT2 expression in the CA1, CA3, and DG hippocampal areas in rats measured 3 d after seizure onset by double-labeling immunofluorescence (yellow arrows indicate neurons; white arrows indicate astroglia-like cells) (scale: 20 μm).

**Figure 8:**
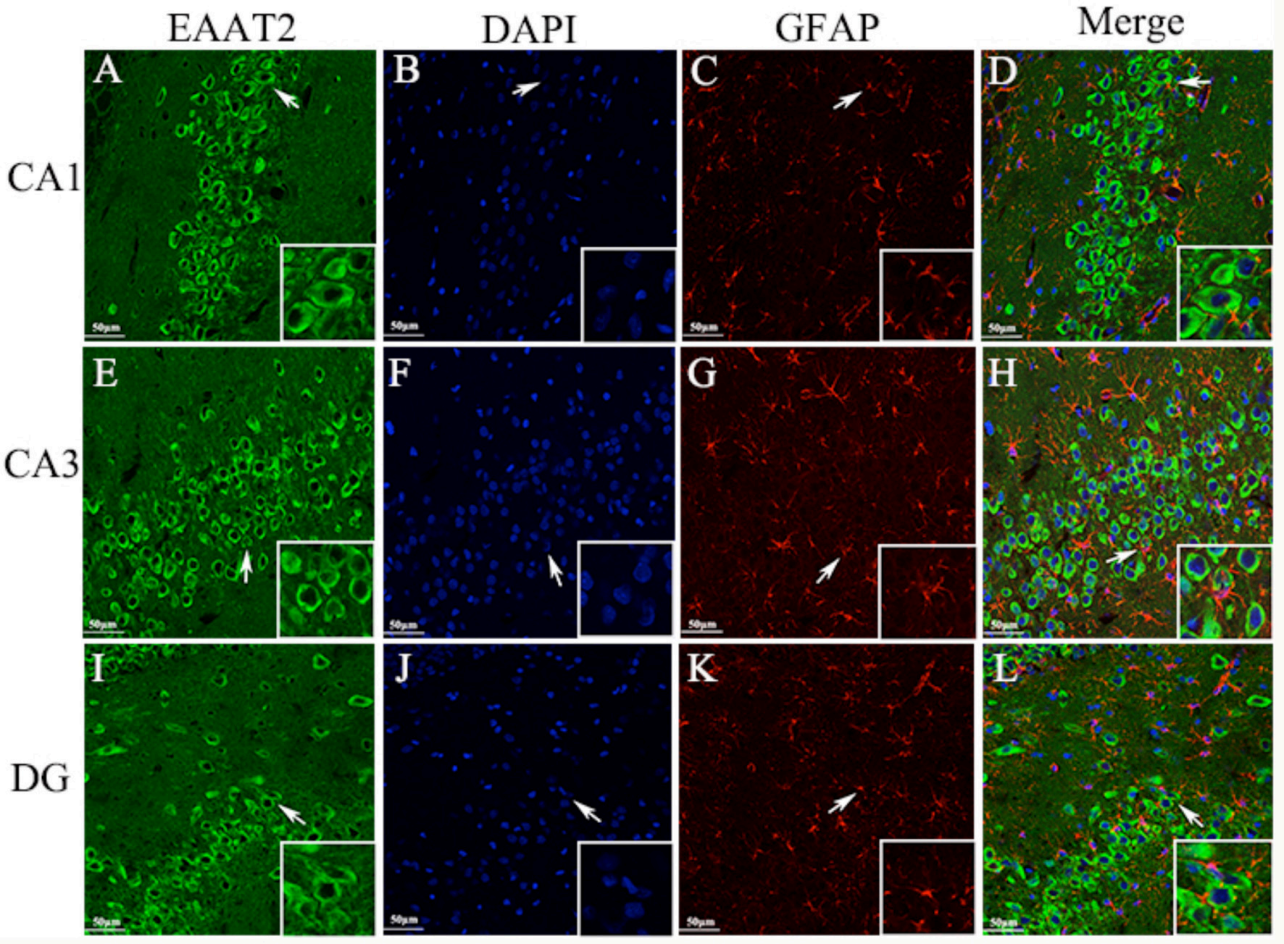
Astroglia **(A–L)**. Same as Figure 7.

Immunohistochemical analyses showed that EAAT2 was expressed mainly in the neuronal cell membrane of the CA1, CA3, and DG hippocampal areas in epileptic rats. To further verify the expression site, we used the double-labeling immunofluorescence technique to accurately locate the expression of EAAT2 in hippocampal tissue cells. Specimens obtained 3 d after seizure were used in the analysis. The results showed that EAAT2, the neuronal dendritic specific marker MAP2 and the astroglia-specific marker GFAP were co-expressed in the CA1, CA3, and DG hippocampal areas of rats. In addition, these proteins were expressed mainly in the cytoplasm and membrane of the positively stained cells. Therefore, EAAT2 is expressed in the hippocampus in neuronal cells and is also expressed in some astroglia (Figures 7, 8).

## Discussion

Epilepsy is a common chronic disease of the nervous system. Although many antiepileptic drugs are used in clinical settings, the control of seizures with drugs remains difficult in ~30% of epilepsy patients because the pathogenesis of epilepsy is unclear; thus, there are no effective means for curing epilepsy (20). EAA, particularly via the excitatory effects of Glu, is thought to play a key role in epileptic seizures. Therefore, the study of the EAAT2 glutamate transporter is of great significance.

In this study, we found that EAAT2 expression in the brain tissue of epileptic rats changed dynamically after the onset of epileptic seizures. The EAAT2 expression levels were increased 24 h, 3 d, and 1 w after seizure onset but then gradually decreased until returning to the normal levels at 2 w. Immunohistochemistry and double-labeling immunofluorescence analyses revealed that EAAT2 was expressed mainly in the cytoplasm and membrane of neurons and astrocytes in the hippocampus of epileptic rats, and these data are consistent with data from previous studies that showed that the EAAT2 glutamate transporter was mainly expressed in the mammalian brain (9). The duration of the first epileptic seizure in rats and their Racine scores were significantly decreased after the administration of SB203580. The expression levels of EAAT2 were also increased, as demonstrated by Western blot and immunohistochemical analyses. We then hypothesized that SB203580 could increase the expression levels of EAAT2 by inhibiting p38 MAPK.

p38 MAPK belongs to the family of stress-activated protein kinases. Stress stimulation (such as ultraviolet light, heat shock, hypertonic environments and protein synthesis inhibitors), cytokines, growth factors, and lipopolysaccharides can activate the p38 MAPK signal transduction pathway (21, 22). SE is the strongest source of CNS stimulation and can cause neuronal ATP depletion, shifts in Ca^2+^, the release of EAA, the production of free radicals and other toxic neuronal reactions, which are effects that lead to the activation of p38 MAPK. ATF-2, NF-AT, and NF-κB can be used as phosphorylation substrates to induce a series of cascade reactions (23). In this study, the expression of T-p38 in brain tissue was significantly downregulated in the SB203580 group compared with the epilepsy group, but no obvious expression of P-p38 was detected in any of the groups. This finding indicates that SB203580 exerts a significant inhibitory effect on p38 MAPK. Therefore, administration of the p38 MAPK inhibitor SB203580 significantly decreased the duration of the first seizure and the Racine scores in rats. These results are consistent with data from previous studies that showed that SB203580 can significantly reduce seizure intensity in a rat model of refractory epilepsy and decrease seizure duration in a rat model of chronic epilepsy (18, 24).

After the onset of SE in rats, the glutamate transporter-1 (GLT1) expression levels in the hippocampus began to increase but returned to the baseline levels 30 d after SE (25). The Western blot results showed that EAAT2 was expressed at basal levels in the control group, but the EAAT2 expression levels were increased after seizure onset, peaking on day 3. These results might be related to the increase in the EAA extracellular concentrations before seizure onset, in the acute seizure stage and at the start of the endogenous protection mechanism. However, with increases in the time after seizure onset (3 d, 1 w, and 2 w), the expression of EAAT2 was gradually restored to the basal levels. However, after SB203580 administration the EAAT2 expression levels increased, and the seizure duration and Racine scores decreased significantly. These results indicated that SB203580 exerted an antiepileptic effect, which might be related to the increase in EAAT2 expression. Previous studies have shown that presynaptic membranes release Glu through exocytosis. This process increases the synaptic concentration of Glu, and the resulting increased concentration causes Ca^2+^ and Na^+^ in neurons to flow internally to excessively excite the neurons. The glutamate transporter EAAT2 can rapidly transport synaptic EAAs from the synaptic cleft. As a major transporter that takes up extracellular Glu, EAAT2 causes 80–90% of these effects (26–30). Thus, EAAT2 plays a very important role in Glu uptake. The increased expression of EAAT2 in brain tissue obtained with SB203580 upregulates Glu uptake, decreases extracellular EAA concentrations and prevents excessive EAA levels from inducing epilepsy. Similarly, increased expression levels of EAAT2 in transgenic mice reduce the number of deaths after SE and reduce neuropathological changes in the hippocampus and seizure progression (31). However, compared with control mice, EAAT2 genetic knockout mice exhibited higher mortality, weight loss and more frequent seizures (32).

In conclusion, EAAT2 plays an important role in epileptic seizures. The expression of EAAT2 can be upregulated by activating transcription or translation (33–36). At the transcriptional level, the EAAT2 promoter can be influenced by most neurochemical signals, such as EGF and TGF-α; these signals increase the transcription of EAAT2, whereas TNF-α inhibits the transcription of EAAT2 (37). In addition, Rothstein and others have found that ceftriaxone can increase the protein levels of EAAT2 by activating transcription (38). At the translational level, some small molecule derivatives and drugs can also activate the translation of EAAT2 to increase the expression of EAAT2 (39–41). Kong and others have found that the compound LDN/OSU-0212320 can affect the translation of EAAT2, increase the expression of EAAT2 in neurons, and increase the uptake of Glu (31). In this study, administration of the p38 MAPK chemical inhibitor SB203580 also increased the expression levels of EAAT2 in the hippocampus and neocortex of the temporal lobe of rats, but the specific regulatory mechanism is unclear and needs further study.

## Author contributions

ZY completed animal models and thesis writing, ZX guided experiment completion and thesis writing, JW and HH completed Western blot, PX and CY completed immunohistochemistry, JZ and YP completed immunofluorescence, ZL and JZ completed Statistical analysis.

### Conflict of interest statement

The authors declare that the research was conducted in the absence of any commercial or financial relationships that could be construed as a potential conflict of interest.
